# Natural selection drives the fine-scale divergence of a coevolutionary arms race involving a long-mouthed weevil and its obligate host plant

**DOI:** 10.1186/1471-2148-9-273

**Published:** 2009-11-27

**Authors:** Hirokazu Toju

**Affiliations:** 1 Research Institute of Genome-based Biofactory, National Institute of Advanced Industrial Science and Technology (AIST), Tsukuba, Ibaraki 305-8566, Japan

## Abstract

**Background:**

One of the major recent advances in evolutionary biology is the recognition that evolutionary interactions between species are substantially differentiated among geographic populations. To date, several authors have revealed natural selection pressures mediating the geographically-divergent processes of coevolution. How local, then, is the geographic structuring of natural selection in coevolutionary systems?

**Results:**

I examined the spatial scale of a "geographic selection mosaic," focusing on a system involving a seed-predatory insect, the camellia weevil (*Curculio camelliae*), and its host plant, the Japanese camellia (*Camellia japonica*). In this system, female weevils excavate camellia fruits with their extremely-long mouthparts to lay eggs into seeds, while camellia seeds are protected by thick pericarps. Quantitative evaluation of natural selection demonstrated that thicker camellia pericarps are significantly favored in some, but not all, populations within a small island (Yakushima Island, Japan; diameter ca. 30 km). At the extreme, camellia populations separated by only several kilometers were subject to different selection pressures. Interestingly, in a population with the thickest pericarps, camellia individuals with intermediate pericarp thickness had relatively high fitness when the potential costs of producing thick pericarps were considered. Also importantly, some parameters of the weevil - camellia interaction such as the severity of seed infestation showed clines along temperature, suggesting the effects of climate on the fine-scale geographic differentiation of the coevolutionary processes.

**Conclusion:**

These results show that natural selection can drive the geographic differentiation of interspecific interactions at surprisingly small spatial scales. Future studies should reveal the evolutionary/ecological outcomes of the "fine scale geographic mosaics" in biological communities.

## Background

Evolutionary biologists have recently acknowledged that interspecific interactions and coevolutionary processes are structured across geographic populations [1-6]. In his geographic mosaic theory of coevolution, Thompson [7,8] argued that the forms and strength of natural selection on interacting species vary among populations (geographic selection mosaic; [9-13]), and therefore reciprocal selection on traits important for interspecific interactions mediates the coevolution of interacting species in some populations (coevolutionary hotspots) but not in others (coevolutionary coldspots). In addition, gene flow, random genetic drift, and extinction of local populations promote the geographic structuring of coevolutionary interactions, sometimes perturbing or promoting the local adaptation of interacting species (trait remixing; [14-17]). Based on this tripartite recognition, the theory predicts that coevolving traits vary among populations [18-25], and that traits are well matched in some local communities but not in others [13,26-29]. Consequently, few coevolving traits or underlying alleles are expected to be widespread across geographic ranges or fixed within interacting species [30].

These geographic structures of coevolution are expected to differ among the spatial scales at which the observations are conducted [3]. Given that the relative contributions of local natural selection and gene flow to the spatial structuring of coevolutionary interactions is dependent on spatial scales, the geographic patterns of local adaptation and maladaptation can vary depending on the spatial scale examined [17]. In addition, because interacting species often differ in their dispersal abilities, the spatial scale of local adaptation can be different between interacting species [31]. Furthermore, unique environmental factors may contribute to the geographic differentiation of coevolutionary interactions at each spatial scale, thereby shaping the spatial hierarchies of coevolutionary processes. Thus, to fully understand the ecological and evolutionary outcomes of the geographic structuring of coevolution, we need to reveal the processes by which coevolutionary interactions of focal systems are geographically differentiated at multiple spatial scales. Nevertheless, there have been few coevolutionary systems in which the geographic variation in coevolving traits, geographic selection mosaics and the effects of gene flow on local adaptation are investigated at more than one spatial scale (cf [32,33]). Moreover, although factors contributing to the geographic differentiation of coevolutionary interactions have been discussed in several interspecific interactions [9,34-36], no study has tested whether such factors could differ, or are the same, among spatial scales.

**Figure 1:**
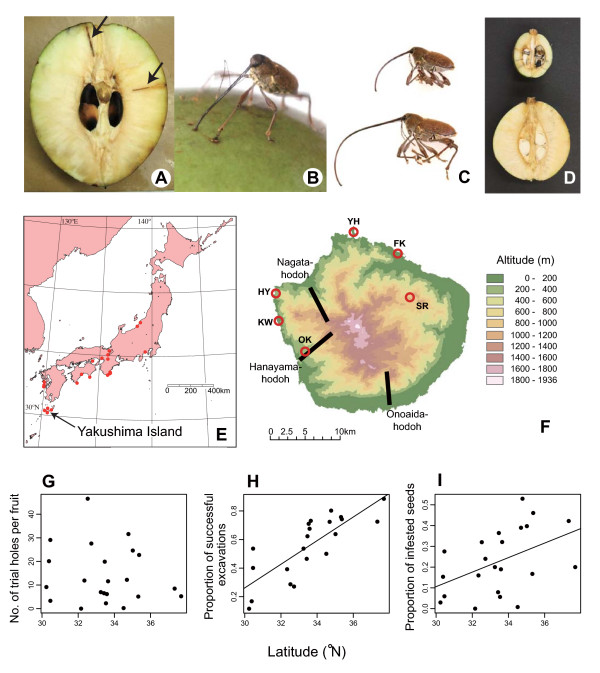
**Study organisms and map of study sites**. (A) Cross-section of a Japanese camellia fruit showing holes in the woody pericarp made by female camellia weevils (arrows). (B) A female camellia weevil drilling with her long rostrum into a camellia pericarp. (C) Geographic variation in weevil rostrum length. Female weevils from Taiji, Honshu (33.58 °N, 135.96 °E; top) and Hanyama, Yakushima Island (30.38 °N, 130.39 °E; bottom). (D) Geographic variation in camellia pericarp thickness. The dissections of camellia fruits in Kiioshima, Honshu (33.47 °N, 135.86 °E; top) and Shitoko, Yakushima Island (30.44 °N, 130.54 °E; bottom). (E) Study sites in which the latitudinal gradient of the ecological interaction between the weevil and camellia was examined (see -G-I). (F) The topography of Yakushima Island. Six populations in which natural selection acting on camellia pericarp thickness was evaluated and three transects used for testing the altitudinal gradients of the weevil-camellia interaction are shown. See Table 1 for abbreviations of populations. (G) The relationship between latitude and the number of trial holes per camellia fruit made by camellia weevils (*y *= - 0.810*x *+ 41.3, *F*_1,20 _= 0.4, *P *= 0.54). See Additional file 1 for sampling localities. (H) The relationship between latitude and the proportion of successful excavations of camellia pericarps by camellia weevils (*y *= 0.0829*x *- 2.22, *F*_1,19 _= 33.1, *P *< 0.0001). (I) Relationship between latitude and the proportion of camellia seeds infested by camellia weevil larvae (*y *= 0.0350*x *- 0.943, *F*_1,20 _= 5.4, *P *= 0.0308).

This paper elucidates the hierarchy of the geographic mosaic of a coevolutionary arms race (sensu [37]) involving a seed predatory insect, the camellia weevil (*Curculio camelliae*: Curculionidae: Coleoptera), and its host plant, the Japanese camellia (*Camellia japonica*: Theaceae) [12,13,28,38,39] (see also [40]) (Fig. 1A, B). The camellia weevil is an obligate seed-predator of the Japanese camellia, whose larvae feed exclusively on camellia seeds. To lay eggs into camellia seeds, which are physically defended by a very thick pericarp, female weevils make holes in the pericarp with their extremely long mouthparts (rostra), into which they insert their ovipositor. In previous studies over a 700-km area in Japan, it was shown that the sizes of the putative coevolving traits, that is, weevil rostrum length and camellia pericarp thickness, varied remarkably between populations (Fig. 1C, D), and that these traits were correlated across the Japanese archipelago [13,28]. Analyses of the geographic variation in natural selection also suggested that both weevil and camellia traits were locally adapted by reciprocal selection between the two species [12,13,39]. In addition, based on population genetic analyses of both species, it was expected that limited gene flow between populations could potentially facilitate geographic differentiation in the coevolutionary processes across the Japanese archipelago [39,41]. Also importantly, latitudinal gradients of the putative coevolving traits (i.e., weevil rostrum length and camellia pericarp thickness), the strength of natural selection on camellia pericarp thickness, and the nature of weevil attacks (Figs. 1G-I) (see additional file 1) [13,39] suggest that climatic factors (e.g., habitat temperature) have promoted the geographic structuring of this coevolutionary arms race [13]. However, the possibility that the ecological and evolutionary interaction between the two species is differentiated at smaller spatial scales (e.g., [42]) has not yet been tested.

To test whether the weevil-camellia coevolutionary interaction is structured at a spatial scale of several kilometers, this study focused on Yakushima Island, in the southernmost region of the area examined in previous studies [12,13,28,38,39] (Fig. 1E). Although small (ca. 30 km diameter), this island has very steep environmental clines in terms of altitude (ca. 1936 m), from coastal subtropical to central cool-temperate areas (Fig. 1F). This variation in environmental conditions is expected to cause geographic divergence of the weevil-camellia coevolutionary processes and thus provide an ideal opportunity to examine "the sizes of the 'tiles' within the geographic mosaic" [3]. A preliminary analysis indicated that weevil rostrum length and camellia pericarp thickness differ significantly among populations separated by only several kilometers on Yakushima [38], and thus high levels of interpopulation variation in natural selection pressures is expected. In addition, analyses of molecular markers for the Japanese camellia and the camellia weevil revealed that significant positive relationships between genetic and geographic distance are observed at scales of 100 m or <10 km, respectively ([43]; reanalysis of data from [39]). Thus, by revealing geographic variation in the direction/strength of natural selection on coevolving traits within Yakushima, one can fully understand the spatial scale of the weevil-camellia arms race and then compare the coevolutionary processes at two spatial scales: within Yakushima Island and across the Japanese archipelago.

Two questions were addressed. First, do the direction and strength of natural selection acting on camellia pericarp thickness vary at the spatial scale of several kilometers? Second, what factors promote the geographic structuring of coevolution within Yakushima Island? I first examined the interpopulation variation in the direction and/or strength of natural selection on camellia pericarp thickness within Yakushima Island using Lande and Arnold's selection analyses [44]. Then, to evaluate the influence of environmental conditions on the geographic mosaic of coevolution, geographic variation in the success and severity of weevil attacks on the Japanese camellia were examined. Furthermore, I tested the hypothesis that climatic factors contribute to the geographic structuring of the weevil-camellia interaction, not only at the entire Japan spatial scale ([13,39]; Figs. 1H, I) but also at a much smaller spatial scale. Overall, the paper shows that coevolutionary processes can be differentiated at surprisingly small spatial scales, and provides a novel example in which the causes of the geographic mosaics of coevolution are investigated at multiple spatial scales.

## Methods

### Study system

The camellia weevil is an obligate predator on seeds of the Japanese camellia, a broad-leaved evergreen tree, common in warm-temperate forests of Japan, southern Korea, and Taiwan. In the wild, the weevil is found over almost the entire range of the Japanese camellia and is an agricultural pest affecting the production of camellia oils [45], although it is usually absent on small islands surrounding the mainlands of Japan. The emergence of the weevil adults usually begins in May, and oviposition occurs when the pericarp of the Japanese camellia has almost matured (usually from mid-July to mid-August; [45]; H. Toju, pers. obs.). Unripe fruits that have yet to reach the maximum size contain small, undeveloped seeds with a jellylike endosperm, which appears to be unsuitable for the development/growth of weevil eggs/larvae [46]. To lay an egg into a camellia seed, a female weevil uses its extremely long rostrum to excavate the very thick, woody pericarp of the camellia (Fig. 1A, B) [46,47]. Importantly, the probability of successful excavation of pericarps increases with increasing weevil rostrum length and decreases with increasing camellia pericarp thickness [13]. Thus, a coevolutionary arms race (sensu [37,48]) is expected to occur between weevil rostrum length and camellia pericarp thickness. Indeed, the length of the camellia weevil rostrum and the thickness of the Japanese camellia pericarp are extraordinary in the weevil genus *Curculio *and the plant genus *Camellia *(see [40,49-52]), suggesting the occurrence of coevolutionary escalation between the two species. The camellia pericarp thickness is assumed to be heritable because an analysis of inter-individual relatedness with microsatellite markers revealed a significant heritability for the trait (H. Toju, H. Abe and S. Ueno, unpubl.; *sensu *[53]). Thus, the plant trait is expected to respond to natural selection exerted by the weevil.

Field sampling over almost the entire range of the two species revealed that both weevil rostrum length and camellia pericarp thickness varied remarkably among populations (9-19 mm for weevils; 6-20 mm for camellias; [13]. Moreover, the sizes of the two traits were clearly correlated across geographic populations, suggesting that at least one of the two species is locally adapted to the other [13,28].

In these previous studies, the geographic selection mosaic for camellia pericarp thickness was evaluated across the Japanese archipelago [12,13]. A quantitative evaluation of natural selection, in which the relationship between camellia pericarp thickness and the proportion of intact (surviving) camellia seeds was estimated in each camellia population, revealed that the direction and strength of natural selection acting on the camellia trait varied significantly between geographic populations [13]. Moreover, thicker camellia pericarps were favored in low-latitude populations [13]. The interpopulation variation in natural selection experienced by camellia was partly attributable to geographic variation in the behavior of female weevils. That is, due to the geographic gradient in the risk of failing to excavate camellia pericarps [13] (Fig. 1G), female weevils tend to avoid attacking camellia fruits with thick pericarps in the low-latitude populations (see [12]). For the camellia weevil, it was suggested that interpopulation variation in rostrum length was mediated by geographically varying pressures of natural selection exerted by the camellia [39]. These studies support the hypothesis that the camellia weevil and the Japanese camellia are involved in a geographically structured coevolutionary arms race [13,28,39].

### Sampling of fruits

Fruits of the Japanese camellia were collected across its entire range on Yakushima Island, Kagoshima Prefecture, Japan (Fig. 1E, F) about 1 month after the oviposition season of the camellia weevil (from August to October 2005, depending on the season of fruit maturation). This circular island is about 30 km in diameter and has a steep climatic cline in altitude, from broad-leaved evergreen forest in lowland areas (0-700 m), mixed broad-leaved evergreen and coniferous forest (700-1200 m), and coniferous forest (1200-1500 m) to bamboo thicket in central areas (1500-1936 m). The Japanese camellia is distributed over the entire island below 1400 m altitude, which is the physiological boundary of the plant. I randomly collected fruits from individual trees (up to two fruits) throughout the island. The sampling locations were recorded using a portable global positioning system unit (Geko 201; Germin Ltd.). Also, the diameter at breast height (DBH) and the number of fruits were recorded for each tree. From those fruit specimens, I excluded fruits without mature seeds and then samples used for subsequent analyses became 968 fruits of 611 individuals from 499 locations. Fruit size was measured to the nearest 0.01 mm using digital calipers; fruit diameter was measured as the mean of a longitudinal diameter and two equatorial diameters that were perpendicular to each other. Pericarp thickness was the mean of four measurements along cross-axes of a longitudinal section of the fruit. Also, whole wet weight and pericarp wet weight were measured for each fruit. These four measurements were averaged for individual trees, respectively. Note that significant variation of pericarp thickness between trees was confirmed in a previous study [13].

### Interpopulation variation

I first focused on camellia individuals from six populations among which significant variation in both camellia pericarp thickness and weevil rostrum length had been found in a previous study [38] to test whether the direction and/or strength of natural selection also varied among the populations (Table 1): geographic distance between populations ranged from 4 to 19 km (Fig. 1F). Note that one population examined previously (Shitoko) was excluded because the small sample size was insufficient for natural selection analyses. In two populations (Yahazu and Hanyama), data on camellia fruit obtained in 2003 [13] were used because almost no fruit was produced in 2005 due to severe salt damage caused by typhoons in the previous year. The results of analyses presented below did not change qualitatively after excluding these two populations. Genetic analyses using 29 microsatellite markers revealed that each of the six populations constituted a unique genetic cluster and gene flow between the populations was limited (Toju et al. unpubl.).

**Table 1:** Six study sites and sample sizes.

				*Curculio camelliae*					*Camellia japonica*							
	**Latitude**	**Longitude**	**Altitude**		**Body length (mm)**		**Rostrum length (mm)**		**No. trees**	**No. fruits**	**No. seeds**	**Fruit diameter (mm)**		**Pericarp thickness (mm)**		**CV**

Locality	(°N)	(°E)	(m)	*N*	Mean	SD	Mean	SD	trees	fruits	seeds	Mean	SD	Mean	SD	(%)

Yahazu (YH)	30.46	130.50	53	20	8.30	0.50	14.54	1.87	41	101	512	48.09	8.08	12.49	3.39	27.2

Fukagawa (FK)	30.44	130.56	12	2	8.16	0.57	12.99	1.14	35	52	271	47.84	8.18	12.97	3.60	27.8

Shiratani (SR)	30.38	130.58	670	2	9.60	0.73	21.11	1.55	33	43	248	58.59	8.86	19.69	3.39	17.2

Hanyama (HY)	30.38	130.39	124	13	9.31	0.68	19.48	1.85	21	51	365	64.87	7.78	20.41	3.99	19.5

Kawahara (KW)	30.35	130.39	147	5	8.87	0.52	18.28	1.61	76	122	702	66.71	6.70	21.21	2.38	10.9

Ohko-rindoh (OK)	30.31	130.42	411	3	9.34	0.16	20.59	0.46	33	56	273	56.49	6.11	19.35	2.68	13.9

Morphological data are from a previous study [38]. The coefficient of variation (CV) of camellia pericarp thickness is also shown.

Before studying interpopulation variation in natural selection pressures exerted on camellia pericarp thickness, I examined the geographic variation in the success and severity of weevil attacks on camellia fruits among the above-mentioned six populations. To evaluate the nature of weevil attacks, I used three variables. First, because the excavations of weevils into camellia pericarps remained as visible holes (Fig. 1A), I counted the number of holes (hereafter, trial holes) for each fruit, and used the number of trial holes per fruit to evaluate the frequency of weevil attacks. Second, in counting the trial holes, I evaluated the success of weevils in reaching seeds and calculated the fraction of the holes reaching seeds to total trial holes for each population, representing the proportion of successful excavations of camellia pericarps by the weevils. Third, I counted the number of seeds within each fruit and recorded the fraction of seeds infested by weevil larvae (hereafter, the proportion of infested seeds): note that each fruit usually contains up to ten seeds. Seeds or ovules that died before maturation were excluded from the total seed number because the mortality factors affecting such seeds, for example, fertilization failure, were difficult to evaluate. Therefore, the mortality of mature seeds were determined exclusively through infestation by weevil larvae. Although Japanese camellia seeds are also rarely attacked by larvae of an unidentified lepidopteran species (Toju, pers. obs.), none of the 4969 seeds examined in 2005 was infested by this species.

For each population, I calculated the number of trial holes per fruit for each tree, and tested its interpopulation variation with the Welch's test and its interpopulation variation in the proportion of successful excavations with the chi-square test. The numbers of successful excavations and of unsuccessful excavations were represented by the number of holes reaching seeds and holes not reaching seeds, respectively. These numbers were pooled within each population, and then a chi-square test was performed. Finally, the chi-square test was also applied to the proportion of infested seeds. Before analysis, the numbers of intact and infested seeds were pooled within each population.

### Relationship between pericarp thickness and the success of weevil attacks

To evaluate the function of thick camellia pericarps in defending against camellia weevils (cf [13]), I examined the relationship between pericarp thickness and the success of weevil attacks for each of the six populations. First, I conducted a regression of the proportion of successful excavations on pericarp thickness. A logistic regression [54] was used to infer the relationship between the success of weevil attacks and the camellia pericarp thickness in each population. To avoid overdispersion, a generalized linear mixed model was used with a logit-link and a binomial error (penalized quasi-likelihood procedure) using R with the MASS package [55]. In the model, individual trees and each fruit nested within trees were fitted as random terms.

I then calculated the pericarp thickness at which the weevil excavation attempts were expected to succeed at a probability of 50% (boring success 50%; *BS*_50_) for each population, with a 95% confidence interval using the delta method [56] (cf., [57]). A population in which a significant relationship between pericarp thickness and the proportion of successful excavations did not exist was excluded from the analysis of the *BS*_50 _(i.e., at Fukagawa).

I expected the *BS*_50 _to be determined by the rostrum length of the sympatric camellia weevils [13] and thus evaluated the relative levels of 'armament escalation' between camellias and weevils based on information from the *BS*_50 _(cf., [13,58]). I postulated that the Japanese camellia was more likely to be successful in defending its seeds against weevil attacks if the plant had already evolved a very thick pericarp relative to the *BS*_50 _and tested this prediction by regressing the proportion of successful excavations on the differences between the mean pericarp thickness and *BS*_50_.

### Quantitative evaluation of natural selection

I evaluated the direction and strength of natural selection on camellia pericarp thickness for each population using Lande and Arnold's selection analyses [44,59]. Two types of fitness measures were used: the "proportion" of surviving (intact) seeds (see [13]) and the "number" of surviving seeds. To obtain the former fitness measure, I counted the number of mature seeds for individual trees and then divided them into two categories, depending on whether they survived or were infested by weevil larvae. Subsequently, I calculated the proportion of surviving seeds for fruit specimens from each tree and used as the fitness measure, after converting this value into relative fitness (i.e., fitness of each individual/mean fitness). The relative fitness was regressed on pericarp thickness, which was *z*-standardized (zero-mean, unit-variance) before regression. Thus, the standardized linear/nonlinear selection coefficients (*β*_*σ *_and *γ*_*σ*_, respectively) [57,60] were obtained for each population. The significance of the linear coefficients was tested by randomization using RT version 2.1 (West, Inc.; http://www.west-inc.com/computer.php). The significance of nonlinear coefficients was tested by nonlinear least-square regressions. In addition, the relationship between pericarp thickness and the proportion of surviving seeds was visualized with a cubic spline [61] using the software glms ver.4.0 [62]. In two populations (Yahazu and Hanyama), data obtained in 2003 [13] were used (see above), which was valid because geographic variation in the direction and strength of natural selection on the camellia trait is usually preserved between years (Toju, unpubl.). Finally, the variance of relative fitness (i.e. opportunity for selection; [59]) was calculated for each population to evaluate the potential maximum strength of natural selection on camellia pericarp thickness.

I then reevaluated natural selection on pericarp thickness based on the number of surviving seeds. This measure is a more standard measure of plant fitness because fecundity of individual plants is determined by the number, but not the fraction, of intact seeds. This measure of fitness was obtained for each tree by multiplying the number of fruits by the average number of surviving seeds in a fruit. To quantify a linear selection coefficient, the number of surviving seeds, which was converted into relative fitness beforehand, was regressed on pericarp thickness (*z*-standardized). Because the number of (surviving) seeds was positively correlated with tree size, as evaluated by DBH, in a population (at Kawahara), DBH (*z*-standardized) was incorporated as an explanatory variable in a generalized linear model (GLM; Gaussian error and identity-link function). A standardized linear selection coefficient for pericarp thickness was obtained as a partial regression coefficient of the GLM for each population. Concomitantly, nonlinear selection coefficients were quantified for the respective populations by quadratic regressions of the relative fitness on the pericarp thickness, in which the effects of DBH were also controlled. R was used for both analyses. Note that excluding DBH from the explanatory variables did not qualitatively change the results. The relationship between pericarp thickness and the number of surviving seeds was visualized with a cubic spline. The opportunity for selection was calculated for each population.

In addition to natural selection analyses for camellia pericarp thickness, the potential resource allocation costs of thick pericarps were examined in the abovementioned four populations. First, the mean wet weight of fruits and that of pericarps were calculated in each population and then geographic variation in each measure was examined with the Welch's test and the Tukey-Kramer's HSD test. Second, the potential tradeoffs between pericarp thickness and the number of fruits produced in a tree was examined by regressing the number of fruits by pericarp thickness across individual trees. The effect of tree size was controlled by incorporating DBH as an explanatory variable in each regression.

### Environmental factors

The latitudinal variation of the nature of weevil attacks (Figs. 1G-I; [13]) suggests that some climatic factors mediate geographic structuring of the coevolutionary processes. In comparison to the geographic variation across the Japanese archipelago, the present study examined the dependence of the weevil-camellia interaction on climates at a much smaller spatial scale, i.e., within Yakushima Island.

First, the geographic pattern of weevil attacks within Yakushima Island was visualized by means of geostatistical analysis. A prediction surface of the geographic variation in the number of trial holes per fruit, calculated for individual trees (*N *= 611), was constructed by kriging, which is a method of the interpolation of a random field and is frequently used in landscape ecology [63]). Using Arc GIS 9 with the Geostatistical Analyst extension (ESRI) according to the user manual, an empirical semivariogram was inferred [64]. Among the models examined (exponential, Gaussian, and spherical), the best-fit model was chosen according to the scores of the root mean square standardized prediction error and the mean prediction error. Isotropy of the semivariogram (i.e. the uniformity in all directions) was assumed because preliminary analyses revealed that models with anisotropy (i.e. directional dependence) did not show good scores for prediction errors. Based on the semivariogram, I computed a prediction surface of the variation in the number of trial holes per fruit within Yakushima Island. This kriging analysis was also applied for the proportion of successful excavations, calculated for individual trees. Trees without trial holes from weevils were excluded from the second analysis; leaving the sample size at 548. Isotropy of the semivariogram was also assumed in this analysis. For the proportion of infested seeds, however, kriging was not performed because preliminary analyses of the semivariogram failed to detect the relationship between geographic distance and difference in this variable, which varied remarkably among neighboring trees.

Second, each number of trial holes per fruit, the proportion of successful excavations of pericarps by weevils, and the proportion of infested camellia seeds was regressed on altitude using R. In addition, regression by annual mean temperature (°C) and annual precipitation (mm) was performed to reveal the effects of climate on the weevil-camellia interaction. The climatic data were obtained from Mesh Climate Data of Japan 2000 [65]. In the regression analyses, generalized linear models were constructed with Gaussian error and identity link functions for the regression of the number of trial holes, and binomial error and a logit link function for that of the remaining two response variables. Taking into account the strong negative correlation between annual mean temperature and annual precipitation in the data set (*r *= - 0.749, *t*_609 _= - 27.9, *P *< 0.0001), univariate regression was performed.

## Results

### Geographic variation in the ecological interaction

**Figure 2:**
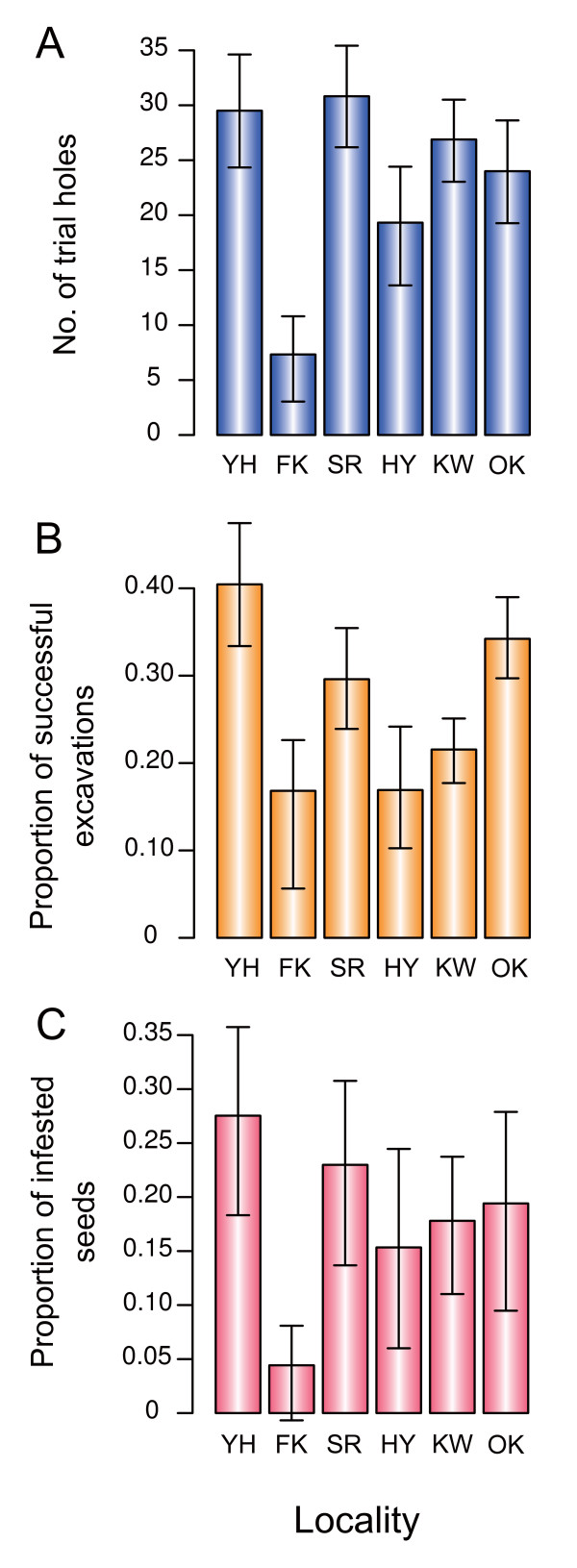
**Interpopulation variation in weevil attacks within Yakushima Island**. (A) Number of trial holes per camellia fruit made by camellia weevils. Bars represent 95% confidence intervals obtained from bootstrap (10,000 replications). (B) Proportion of successful excavations of camellia pericarps by camellia weevils. (C) Proportion of camellia seeds infested by camellia weevil larvae.

The number of weevil attacks per camellia fruit varied significantly between the six populations examined (Welch's test; *F*_5,165.2 _= 17.5, *P *< 0.0001). Among the populations, the camellia population at Fukagawa was subject to less frequent attacks (Fig. 2A). The proportion of successful excavations of camellia pericarps by weevils significantly differed between populations (*χ*^2 ^= 216.7, df = 5, *P *< 0.0001). In Fukagawa, Hanyama, and Kawahara, only one of five attacks by weevils was successful (Fig. 2B). The probability of the success of weevil attacks in these populations on Yakushima Island was the lowest among the previously examined populations across the Japanese archipelago (Fig. 1H). The severity of seed infestation by weevil larvae (i.e., the proportion of infested seeds) also varied among populations within Yakushima Island (*χ*^2 ^= 68.7, df = 5, *P *< 0.0001). The proportion of infested seeds was especially low in Fukagawa (Fig. 2C), where the frequency of weevil attacks was lowest (Fig. 2A).

### Relationship between pericarp thickness and the success of weevil attacks

**Figure 3:**
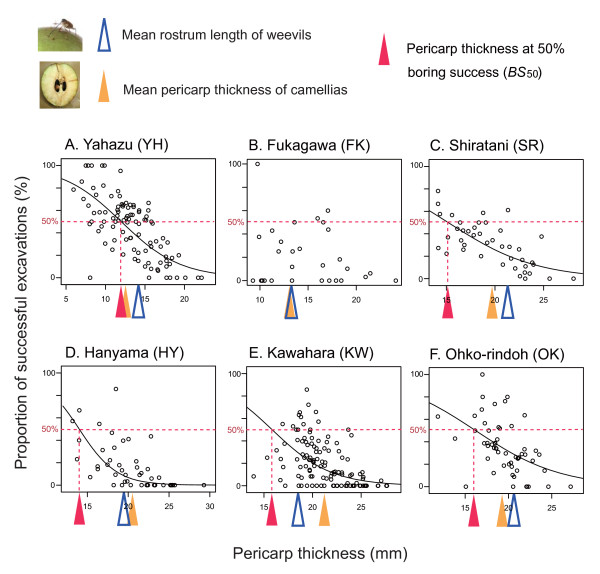
**Relationship between camellia pericarp thickness and the success of weevil attacks**. Solid line indicates a significant relationship between the pericarp thickness of the Japanese camellia and the proportion of successful excavations by the camellia weevil (logistic regression; see Table 2). Triangles represent mean rostrum length of camellia weevils (open blue), mean pericarp thickness of camellias (filled yellow), and the pericarp thickness at which weevil excavation attempts are expected to succeed at a probability of 50% (*BS*_50_; filled red).

**Figure 4:**
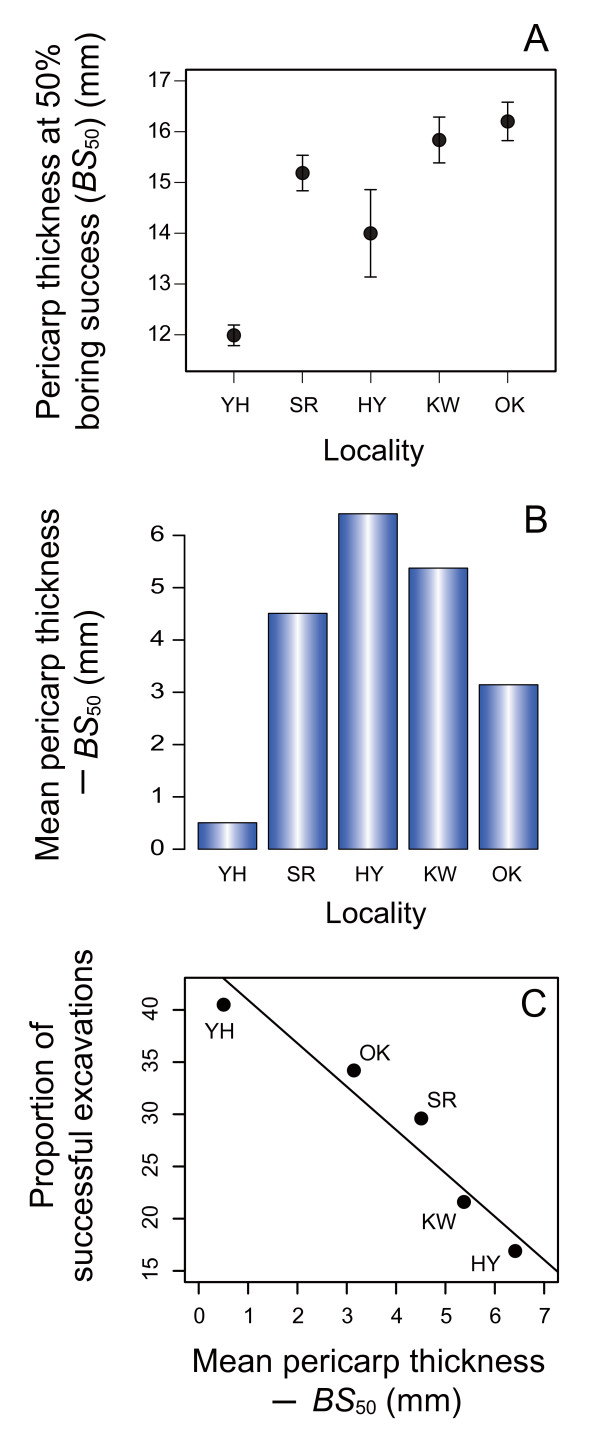
**Interpopulation variation in the camellia pericarp thickness at which a half of weevil attacks are successful**. (A) In each population, the pericarp thickness at which weevil excavation attempts are expected to succeed at a probability of 50% (*BS*_50_; ± 95% CI) was calculated from a logistic regression (Fig. 3; Table 2). A population (Fukagawa) was excluded due to lack of a significant relationship between camellia pericarp thickness and the success of weevil attacks. (B) Difference between mean pericarp thickness and *BS*_50_. (C) Relationship between the degree of pericarp evolution and the success of weevil attacks. The proportion of successful excavations of camellia pericarps by camellia weevils was regressed on the difference between mean pericarp thickness and *BS*_50_. Solid line represents a significant reduced major axis regression.

A clear relationship was observed between camellia pericarp thickness and the probability of successful excavations of pericarps by weevils in five of the six populations examined (Fig. 3; Table 2). This indicates that thicker camellia pericarps were effective at defending seeds from weevils in these plant populations. Note that the pericarp thickness at which the probability of successful excavations was 50% (i.e., *BS*_50_) varied significantly among populations (Fig. 4A; compare 95% confidence intervals). To avoid the attacks of weevils by a probability of 50%, a 12-mm-thick pericarp is sufficient in Yahazu, whereas a 16-mm-thick pericarp is needed in Ohko-rindoh (Fig. 4A). Due to variations in *BS*_50 _and pericarp thickness (Table 1), interpopulation variation occurred in the difference between mean pericarp thickness and *BS*_50 _(Fig. 4B). This result indicates that some populations of Japanese camellia have already evolved pericarps thick enough to defend the seeds against most camellia weevil attacks (e.g., Hanyama and Kawahara in Fig. 4C), while other populations have not (e.g., Yahazu). As expected, weevils were more susceptible to failure of attacks in populations in which the difference between mean pericarp thickness and *BS*_50 _(mean - *BS*_50_) was larger (*y *= -4.15*x *+ 45.1, *N *= 5, *r *= -0.97, *P *= 0.0078; reduced major axis regression; Fig. 4C).

**Table 2:** Relationship between camellia pericarp thickness and the success of weevil attacks.

Locality	No. trial holes	**Coef**.	SE	*t*	*P*
Yahazu (YH)	2912	- *0.268*	0.031	- *8.6*	< 0.0001*

Fukagawa (FK)	416	- *0.149*	0.087	- *1.7*	0.1485

Shiratani (SR)	1311	- *0.221*	0.037	- *5.9*	0.0002*

Hanyama (HY)	1017	- *0.437*	0.059	- *7.4*	< 0.0001*

Kawahara (KW)	3262	- *0.327*	0.038	- *8.5*	< 0.0001*

Ohkorindo (OK)	1312	- *0.204*	0.051	- *4.0*	0.0006*

The proportion of successful excavations of camellia pericarps by camellia weevils was regressed on camellia pericarp thickness for each population. In each model, generalized linear mixed model with binomial error and a logit-link function was constructed. Individual camellia trees and each fruit nested within trees were fitted as random terms to avoid overdispersion. Trees with no trial holes were excluded from the analysis.

*Significant after sequential Bonferroni correction (*α *= 0.05).

### Quantitative evaluation of natural selection

**Figure 5:**
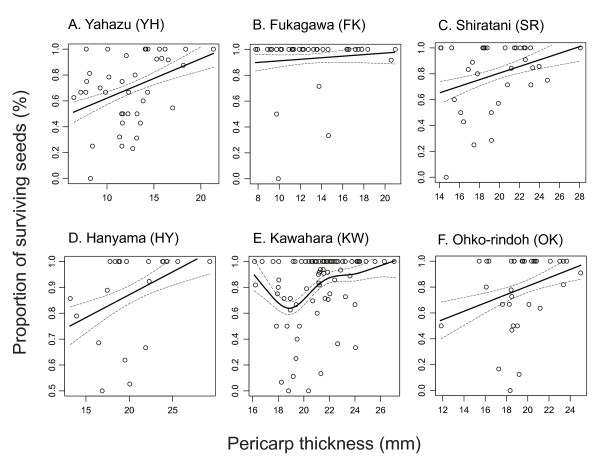
**Form of natural selection evaluated based on the proportion of surviving seeds**. The proportion of surviving seeds was regressed on pericarp thickness in each population (see Table 3). Solid lines represent prediction curves by cubic splines with ± SE.

Natural selection analyses, in which the proportion of surviving seeds was used as a fitness measure, revealed significant or marginally significant directional selection for thicker pericarps in four of the six populations examined (*P *< 0.09), whereas it was nonsignificant but positive in the remaining population (Fukagawa; *P *> 0.5) (Table 3). Although cubic spline visualization showed a U-shaped fitness function in Kawahara (Fig. 5), significant disruptive or stabilizing selection was not observed in any populations (Table 3).

**Table 3:** Relationship between camellia pericarp thickness and the proportion of surviving seeds.

	Opportunity for	*β*_*σ*_				*γ*_*σ*_			
**Locality**	**selection**	**Coef**.	**SE**	***t***	***P***	**Coef**.	**SE**	***t***	***P***

Yahazu^*a *^(YH)	0.152	0.149	0.058	2.6	0.0137	0.111	0.089	1.2	0.2224

Fukagawa (FK)	0.055	0.024	0.040	0.6	0.5781	0.026	0.079	0.3	0.7459

Shiratani (SR)	0.111	0.109	0.057	1.9	0.0639	0.004	0.097	0.0	0.9660

Hanyama^*a *^(HY)	0.039	0.080	0.041	1.9	0.0685	0.021	0.069	0.3	0.7643

Kawahara (KW)	0.114	0.100	0.038	2.7	0.0094	0.038	0.055	0.7	0.4956

Ohko-rindoh (OK)	0.134	0.111	0.063	1.8	0.0878	0.038	0.080	0.5	0.6398

The proportion of surviving (intact) seeds was regressed on camellia pericarp thickness for each population. Standardized linear/nonlinear selection coefficients [57,60] are shown (*β*_*σ *_and *γ*_*σ*_, respectively) with the opportunity for selection (i.e. the variance of relative fitness; [44]).

^*a*^Data from a previous study [12].

**Figure 6:**
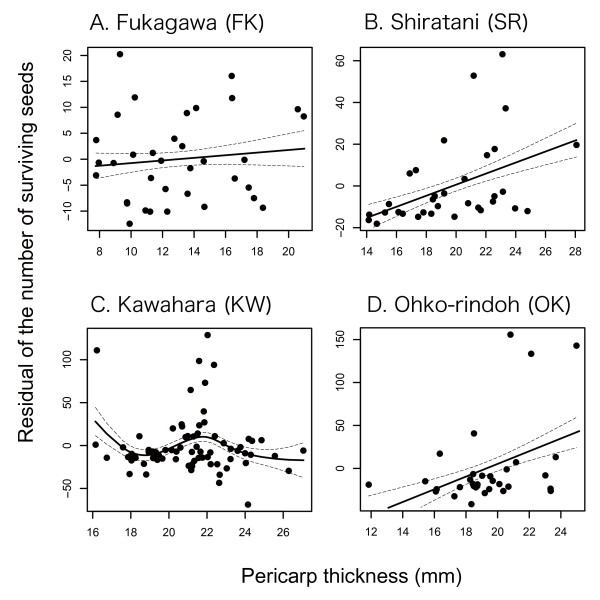
**Geographic selection mosaic for camellia pericarp thickness**. The number of surviving seeds was regressed on pericarp thickness in each population (see Table 4 and Additional file 2). Because the effect of individual tree size, as evaluated by diameter at breast height, was controlled in the regression, the residual of the number of surviving seeds is shown in this figure. Solid lines represent prediction curves by cubic splines with ± SE.

I then reevaluated natural selection on camellia pericarp thickness using another fitness measure, the number of surviving seeds. In Shiratani and Ohko-rindoh, in which marginally significant directional selection for thicker pericarps was detected in a previous natural selection analysis (Table 3), thicker pericarps were favored (Table 4; Fig. 6). In Kawahara, however, natural selection for thicker pericarps was not confirmed (compare Table 4 with Table 3); note that an outlier individual was excluded from this analysis. No relationship between pericarp thickness and fitness was observed in Fukagawa, where the opportunity for selection was the smallest among the four populations analyzed (Table 4). I found no evidence of nonlinear selection acting on this plant trait in the four populations (Additional file 2). However, individuals with intermediate pericarp thickness had relatively high fitness in Kawahara (Fig. 6).

**Table 4:** Geographic variation in natural selection acting on camellia pericarp thickness.

		Pericarp thickness (*β*_*σ*_)				Pericarp thickness (*β*_*σ*_)			
		
Locality	Opportunity for selection	**Coef**.	SE	*t*	*P*	**Coef**.	SE	*t*	*P*
Fukagawa (FK)	0.459	0.074	0.119	0.6	0.5380	- *0.024*	0.119	- *0.2*	0.8420

Shiratani (SR)	1.305	0.524	0.188	2.8	0.0089*	0.141	0.188	0.8	0.4566

Kawahara (KW)	1.998	- *0.070*	0.147	- *0.5*	0.6340	0.660	0.147	4.5	< 0.0001*

Ohko-rindoh (OK)	2.764	0.669	0.277	2.4	0.0218	0.136	0.277	0.5	0.6265

The number of surviving seeds, which was converted into relative fitness, was regressed on the pericarp thickness of camellia trees for each population. The effect of tree sizes on the number of seeds was controlled for by incorporating diameter at breast height (DBH) into a generalized linear model (i.e. multiple regression on pericarp thickness and DBH). Both explanatory variables were *z*-standardized (zero-mean, unit-variance) before regression. Thus, the partial regression coefficient for pericarp thickness represents standardized linear selection coefficient (i.e. *β*_*σ*_). The opportunity for selection (i.e. the variance of relative fitness) is also shown. See additional file 2 for the results of quadratic regression analyses.

*Significant after sequential Bonferroni correction, which was applied for each column (i.e. explanatory variable) (*α *= 0.05).

Among the four populations examined above, camellias in Kawahara had the heaviest fruits (Welch's test; *F*_3,80.8 _=, *P *< 0.0001; Tukey-Kramer's HSD test; *α *= 0.05, Fukagawa < Ohko-rindoh ≈ Shiratani < Kawahara: Table 5) and pericarps (Welch's test; *F*_3,80.4 _=, *P *< 0.0001; Tukey-Kramer's HSD test; *α *= 0.05, Fukagawa < Ohko-rindoh ≈ Shiratani < Kawahara: Table 5) on average. Importantly, the number of fruits decreased with increasing pericarp thickness across trees examined in the natural selection analysis in Kawahara, although the relationship was marginally significant (Table 5). The negative correlation between fruit production and pericarp thickness was not observed in other populations (Table 5).

**Table 5:** Costs of thick camellia pericarps.

					Regression: No. of fruits							
	**Fruit weight (g)**		**Pericarp weight (g)**		**Pericarp thickness**				**DBH**			

Locality	Mean	SD	Mean	SD	Coef.	SE	*t*	*P*	Coef.	SE	*t*	*P*

Fukagawa (FK)	61.71	38.28	55.51	37.05	0.194	0.164	1.2	0.2448	0.342	0.164	2.1	0.0442

Shiratani (SR)	107.30	49.24	103.29	47.78	0.086	0.181	0.5	0.6380	0.186	0.181	1.0	0.313

Kawahara (KW)	156.60	46.20	149.31	43.95	- *0.169*	0.101	- *1.7*	0.0981	0.503	0.101	5.0	< 0.0001

Ohko-rindoh (OK)	95.46	29.80	92.47	29.07	0.214	0.174	1.2	0.2290	0.207	0.174	1.2	0.243

The mean weight of camellia fruits and pericarps are shown for each locality. The potential tradeoff between pericarp thickness and the number of fruits were tested by the regression analyses, in which individual tree size (i.e. DBH) was controlled. All response and explanatory variables were *z*-standardized before regression analyses.

### Environmental factors

**Figure 7:**
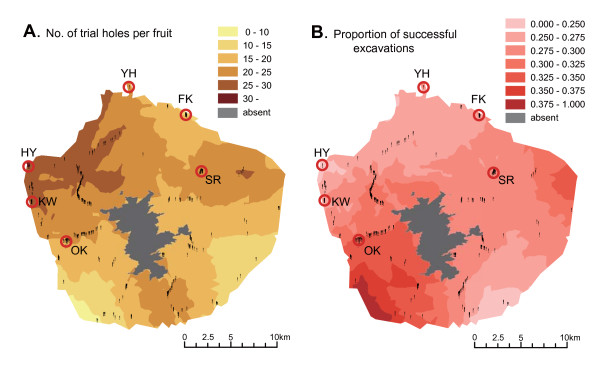
**Geostatistical analyses of weevil attacks on camellia fruits within Yakushima Island**. (A) Geographic variation in the number of trial holes per camellia fruit made by camellia weevils. Areas where the Japanese camellia is absent (> 1,400 m) are represented by grey. Vertical bars represent sampling locations. (B) The geographic variation in the proportion of successful excavations of camellia pericarps by camellia weevils.

A geostatistical analysis revealed that the frequency of weevil attacks (i.e., the number of trial holes per fruit) varied locally within Yakushima Island (Fig. 7A; Gaussian model), as shown in Fig. 2A. In the northwest part of the island, camellia fruit was subjected to frequent weevil attacks (<25 times per fruit), whereas in the southwestern area, fruits were only attacked a few times (Fig. 7A). The probability of the success of weevil attacks (i.e., the proportion of successful excavations) also varied within the island (Fig. 7B; Gaussian model). In the southwest, the excavations of camellia pericarps by weevils were more likely to succeed, whereas in the north and southeast most weevil attacks failed (Fig. 7B).

Regression analyses further revealed geographic patterns of the nature of weevil attacks. The proportion of successful excavations and the proportion of infested seeds increased at higher altitudes (Table 6). As expected by the altitudinal gradient, the two variables were significantly increased with decreasing annual mean temperature (Table 6). All of the number of trial holes per fruit, the proportion of successful excavations, and the proportion of infested seeds increased with increasing annual precipitation (Table 6; marginally significant for the number of trial holes), respectively.

**Table 6:** Effects of climate on the geographic variation in weevil attacks within Yakushima Island.

	No. of trial holes per fruit^*a*, *b*^				Proportion of successful excavations^*c*^				Proportion of infested seeds^*a*^			
	
Locality	Coef. (SE)	**d.f**.	*t*	*P*	Coef. (SE)	**d.f**.	*t*	*P*	Coef. (SE)	**d.f**.	*t*	*P*
Altitude	0.014 (0.041)	609	0.3	0.73	0.189 (0.018)	546	10.6	< 0.0001*	0.403 (0.038)	609	10.7	< 0.0001*

Temperature	0.028 (0.041)	609	0.7	0.489	- 0.166	546	- *9.6*	< 0.0001*	- 0.326	609	- *8.8*	< 0.0001*

Precipitation	0.079 (0.040)	609	2.0	0.051	0.162 (0.017)	546	9.5	< 0.0001*	0.335 (0.040)	609	8.4	< 0.0001*

A generalized linear model was constructed to explain the geographic variation in each of the number of trial holes per fruit, the proportion of successful excavations of camellia pericarps by weevils and the proportion of seeds infested by weevil larvae. An univariate regression was performed for each explanatory variable, annual mean temperature (°C), annual precipitation (mm), the annual mean of the monthly amount of global solar radiation with shading effects of topography (MJ/m^2^). All explanatory variables and the number of trial holes were *z*-standardized (zero-mean, unit-variance).

*Significant after sequential Bonferroni corrections applied for each response variable.

^*a*^No. of trees = 611, no. of fruits = 968.

^*b*^The response variable was *z*-standardized.

^*c*^No. of trees = 548, no. of fruits = 844.

## Discussion

A series of selection analyses showed herein that natural selection driving interspecific interactions could vary among geographic populations separated by only several kilometers. Results further suggested that interpopulation variation in trait costs or climatic environment could promote the geographic differentiation of coevolutionary processes. Thus, these findings detailed below illuminate the possibility that evolutionary interactions between species are spatially far more dynamic than ever thought.

### Potential of a coevolutionary arms race

Before considering weevil attacks as a major driving force of pericarp evolution, alternative factors that may also promote the evolution of thick pericarps should be considered. Importantly, camellia pericarps dehisce after seed maturation and seeds then fall to the ground; hence, pericarps are not an adaptation for attracting animals to disperse seeds (see [66]). Therefore, the adaptive function of pericarps is likely to be protecting seeds against abiotic or biotic environmental factors before seed dispersal. For example, desiccation or cold/frost damage to seeds might favor thicker pericarps. However, given that a sympatric congener of the Japanese camellia, *Camellia sasanqua*, bears fruit with a very thin pericarp (ca. 1 mm), thick pericarps should not be necessary to defend Japanese camellia seeds against abiotic environmental stresses. In addition, thicker pericarps are found in regions at lower latitudes or lower altitudes, where climatic conditions are relatively moderate [13,38]. Alternatively, the evolution of thick pericarps may be driven by organisms that attack camellia seeds. Apart from weevils, the larvae of an unidentified lepidopteran species infest camellia seeds very rarely; however, its impact on the evolution of pericarp thickness is thought to be negligible (see Methods).

In contrast to abovementioned alternatives, the adaptive significance of having thick pericarps to defend against the camellia weevil is evident from the results. These clearly showed that the camellia weevil is a very important mortality factor for camellia seeds (Fig. 2C), and that camellias defend against weevil attacks, that is, excavation of pericarps, more effectively when they have thicker pericarps (Fig. 3; Table 2). Because this negative relationship between camellia pericarp thickness and the probability of successful weevil attacks has also been supported by a laboratory experiment [13], it is highly probable that camellias have evolved thicker pericarps to defend their seeds against weevils. Indeed, the proportion of surviving camellia seeds, which represents fitness of camellia individuals, was found to be positively correlated with pericarp thickness within Yakushima Island (Table 3). It is also noteworthy that the Japanese camellia has significantly thicker pericarps on islands with the camellia weevil than on islands where the weevil is absent (H. Toju, H. Abe and S. Ueno, unpubl.). Overall, these results indicate that defending seeds against the camellia weevil is the most plausible explanation for the evolution of thick camellia pericarps. In the following discussion, I postulate that the pericarp thickness of the Japanese camellia can evolve through natural selection exerted by the camellia weevil.

### Spatial scale of the geographic selection mosaic

Previous studies [13,28,38] have shown that camellia pericarp thickness and weevil rostrum length varied remarkably within Yakushima Island (Table 1). At the extreme, the camellia and weevil traits were differentiated between populations separated by only several kilometers, and the sizes of these defensive and offensive traits were correlated across populations within the island [38]. In addition to phenotypic variation, the success and severity of weevil attacks on camellias varied geographically within Yakushima Island (Figs. 2 and 7). These results suggest that the evolutionary interaction between the two species also varies at the same spatial scale.

The results first indicate that the benefit of having thicker pericarps in resisting severe weevil attacks was ubiquitous among camellia populations within Yakushima Island. In most populations, excluding Fukagawa, where the sample size of trial holes was small (Fig. 2), thicker pericarps were effective in defending against weevil excavations (Fig. 3). In addition, in the same five populations, camellia individuals with thicker pericarps were more likely to protect seeds from oviposition of weevils (Table 3; Fig. 5).

Nonetheless, several lines of evidence indicate that the strength of directional selection for thicker pericarps varies geographically. First, the camellia pericarp thickness at which weevil excavations are successful by the probability of 50% (i.e., *BS*_50_) differed among populations (Fig. 4A), suggesting that the degree of pericarp evolution necessary to defend against weevils is geographically differentiated. This is presumably due to differences in the mean rostrum length of sympatric female weevils (Fig. 3). Second, natural selection analyses showed that camellias with thicker pericarp were favoured in five populations, while no significant selection was observed in the remaining population, Fukagawa (Table 3).

It is important to note that the evolution of thick pericarps may be restricted by the cost of having such a trait, despite the clear benefit of thick pericarps in defending against attacks by camellia weevils. For example, if the amount of photosynthetic products is limited, producing thick pericarps may reduce the number of fruits or seeds that can be produced. Therefore, I examined the geographic variation in natural selection pressures, using the number of surviving seeds as a measure of fitness. This should incorporate the effects of potential tradeoffs between the pericarp thickness of individual fruits and the number of fruits produced. Quantitative evaluation of natural selection indicated that natural selection pressures exerted on camellia pericarp thickness varied within Yakushima Island (Fig. 6; Table 4). Relatively strong directional selection (*β*_*σ *_> 0.5; cf., [67]) was detected for pericarp thickness in Shiratani and Ohko-rindoh, while thicker pericarps were not favored in Kawahara and Fukagawa. Because camellias have considerably heavy pericarps in Kawahara compared to those in other populations (Table 5), producing pericarps would be more costly in the population. Indeed, the number of fruits produced by camellia individuals decreased with increasing pericarp thickness in Kawahara, although the correlation was marginally significant (Table 5). On the other hand, much less resources were allocated to pericarps in Fukagawa compared to other populations and the resource costs of producing pericarps may not explain nonsignificant directional selection observed in this population (Tables 4 and 5). Alternatively, the low frequency of weevil attacks (Fig. 2) and the resultant lack of benefit of having thick pericarps in this population (Fig. 5; Table 3; see also the opportunity for selection in Table 5) would be responsible for the lack of significant directional selection (Table 4; Fig. 6). Overall, these results indicate that the (co-)evolutionary process of the weevil-camellia interaction is structured at a spatial scale of several kilometers (e.g., Ohko-rindoh vs. Kawahara), as suggested by the phenotypic variations in the weevil and camellia traits within this island.

The extent of local adaptation, however, is influenced not only by local selection pressures but also by the levels of gene flow for both species. For the Japanese camellia, gene flow is expected to be localized at fine spatial scales because the home range of its major avian pollinator (*Zosterops japonica*; [68]) is estimated to be 0.3 ha [69], and seed dispersal by rodent dispersers is only by 5.8 m on average [66]. Indeed, population genetic analyses using microsatellite markers have shown a significant positive relationship between genetic and geographic distance (i.e., isolation by distance) between adult trees at spatial scales of 100 m ([43]; see also [41,70-72]). In addition, for weevils, analysis of molecular variance [73] from the published data on mitochondrial DNA sequences [39] have revealed significant genetic differentiation in the six populations examined in this study (H. Toju and T. Sota, unpubl.). Pairwise interpopulation comparison of mitochondrial haplotypes of the weevils also indicated that gene flow is restricted, even between populations separated by only several kilometers (e.g., Fukagawa vs. Shiratani; H. Toju and T. Sota, unpubl.). Consequently, the evidence for localized gene flow for both species suggests that geographic selection mosaics can drive fine-scale phenotypic differentiation of camellia pericarp thickness and weevil rostrum length among populations, in spite of the homogenizing effects of gene flow (see [17,74]).

### Relationship between natural selection and levels of coevolutionary escalation

Although the geographic selection mosaic for camellia pericarp thickness indicates that the process of weevil-camellia coevolution is spatially structured, variation in the levels of coevolutionary escalation, as evaluated by camellia pericarp thickness and weevil rostrum length, were not consistent with variations in current selection pressures. That is, significant directional selection for thicker pericarps was not detected in Kawahara (Table 4; Fig. 6), in which the camellia and weevil traits have been already highly escalated (Table 1). The present direction and/or strength of natural selection, however, are not necessarily the same as those in the past [75]. Given that the volume or weight of resources allocated to pericarps is proportional to cubed pericarp thickness, evolving per unit thickness of pericarps is expected to become increasingly costly as coevolutionary escalation proceeds. Therefore, although the benefit of having thicker pericarps is still significant in Kawahara (Table 3), considerable costs of resource allocation to pericarps may prevent further evolution (Tables 4 and 5). In fact, a cubic spline analysis showed that camellia individuals with intermediate pericarp thickness represent relatively high fitness as balanced by the benefit (Table 3) and cost (Table 5) of having thick pericarps, although relatively small sample size might prevent the detection of significant stabilizing selection (Additional file 2; [67]). Thus, the coevolutionary process may be at an equilibrium in Kawahara, whereas in Shiratani and Ohko-rindoh, relatively low resource costs may allow the evolution of thicker pericarps (Fig. 6; Table 5).

In addition to trait costs, two other factors may cause the negative or positive relationships between the levels of coevolutionary escalation and the strength of directional selection for thicker pericarps. One is suggested by the interpopulation variation in the difference between mean pericarp thickness and *BS*_50 _(Fig. 4B). Because camellias have already evolved very thick pericarps to resist most weevil attacks in some populations (e.g., Hanyama and Kawahara; Figs. 3 and 4C), further evolution of pericarps may not effectively increase fitness in these camellia populations. This loss of the benefit of thicker pericarps, however, is not evident in the present data sets (see Kawahara in Table 3). The second factor is the dependence of weevil behavior, that is, the preference of female weevils for camellia fruits based on pericarp thickness, on the levels of coevolutionary escalation [12]. A previous study showed that the preference of weevils varied among populations, and that interpopulation variation in weevil behavior is an important determinant of the geographic selection mosaic for camellia pericarp thickness [12]. Importantly, female weevils avoid attacking fruits with relatively thick pericarps in populations in which they are subject to higher risks of failure in attacking such fruits due to low average probability of successful excavations [12]. Given that the proportion of successful excavations decreases as coevolutionary escalation proceeds ([13]; see also Fig. 4C; Table 1), presumably due to the mortality costs of weevils with long rostra (cf., [76]) and the resultant limitation in the evolution of this weevil trait, thicker pericarps might further increase the fitness of camellias. Indeed, camellia fruits with relatively thick pericarps were avoided by weevils in Ohko-rindoh and Shiratani (Toju, unpubl.), leading to further directional selection (Table 4), but were preferred in Fukagawa, preventing the evolution of thick pericarps. No significant tendency was observed in Kawahara.

### Environmental dependence of the weevil-camellia coevolution

Geostatistical analyses confirmed that the attacks of weevils on camellia fruits varied at a fine scale within Yakushima Island (Fig. 7). Moreover, regression analyses showed that the proportion of successful pericarp excavations by weevils and the proportion of infested seeds increased with decreasing temperature within the island (Table 6).

The environmental clines of the weevil-camellia interaction are observed not only within Yakushima Island, but also across the Japanese archipelago [13,28,39]. Examinations of altitudinal gradients within this island, and latitudinal gradients over the whole of Japan, have revealed that the proportion of successful excavations and the proportion of infested seeds increase in the cooler-temperate regions (Table 6; Fig. 1G, I; [13]). Analyses at within-Yakushima and entire-Japan scales also showed that the size of the camellia defensive trait, i.e. pericarp thickness, decreased with increasing altitude and latitude [13,28,38,39]. Together, these results suggest that climatic factors (e.g., low temperature) limit the evolution of thick camellia pericarps in populations at higher altitudes and latitudes, despite the more severe impacts of seed infestation by weevils in these areas.

A putative factor responsible for the geographic differentiation is geographic variation in "productivity." Mathematical models of predator-prey or parasitoid-host coevolution predict that environmental factors influencing the fecundity, density-dependent factors limiting population dynamics, and the costs of defensive traits of victims determine the occurrence of arms races or the levels of coevolutionary escalation ([77-79]; N. Iseki, H. Toju and A. Sasaki, unpubl.). Although these predictions have not yet been demonstrated in the wild (cf., the laboratory study of [14]), the influence of productivity on local coevolutionary dynamics [35,80] is expected in the weevil-camellia system. For example, because the photosynthetic capacity of camellias increases exponentially with increasing temperature [81], local climate would affect the optimal resource allocation strategy [82] (*sensu *[83]) or fecundity of camellias, thereby leading to the spatial variation in the levels of coevolutionary escalation (N. Iseki, H. Toju and A. Sasaki, unpubl.). Consequently, findings in the weevil-camellia system support the hypothesis that spatial variation in climate is a major factor driving the geographic differentiation of ecological and evolutionary interactions between species [84-87].

In contrast to the potential dependence of the weevil-camellia interaction on temperature, the relationship between annual precipitation and the variables representing weevil attacks (Table 6) was not straightforward. Because the mean of annual precipitation experienced by sampled individuals is very high (3809.9 mm; SD = 297.0 mm), the photosynthetic activity of the Japanese camellia is unlikely to be affected by the availability of water. Thus, given the negative correlation between annual mean temperature and annual precipitation (see Methods), the association between precipitation and the nature of weevil attacks may be a statistical artifact.

## Conclusion

The results of this study show that ecological and evolutionary interactions between the camellia weevil and the Japanese camellia are structured at a surprisingly fine spatial scale, i.e., within several kilometers. Thus, the spatial scale of geographic structures of coevolution can be very small (cf [42]). However, it is expected that the "sizes of the 'tiles' within geographic mosaics" [3] vary, depending on the characteristics of focal coevolving systems. Potential factors determining the spatial scale of the geographic mosaics involve a balance between local natural selection and levels of gene flow, which is expected to differ among systems, depending on the migration abilities of the interacting species [88]. Furthermore, the direction and strength of local natural selection *per se *can be affected by the pattern of migration (i.e. migration load; [89]), influencing the spatial scale of geographic selection mosaics. Therefore, to further clarify the mechanisms mediating the geographic structuring of coevolutionary interactions, comparative studies should be conducted between systems in which natural selection, gene flow, and local adaptation are investigated simultaneously.

In this study, I examined the environmental factors mediating the geographic structuring of the weevil-camellia coevolutionary process at a small spatial scale, in comparison with previous analyses at a larger spatial scale [13,28,39]. To date, several studies have reported that the species composition of local communities (e.g., [9,34,36,90,91]) or spatial variation in climate (e.g., [13,92]) has contributed to the geographic differentiation in coevolutionary interactions. Such mechanisms mediating the spatial process of coevolution, however, can differ between spatial scales. Alternatively, if the same factor is suggested to play a major role in shaping the geographic mosaic of coevolution at multiple spatial scales of a system, the importance of that focal factor will be further emphasized. Hence, we need to properly understand the spatial scales at which focal factors drive the geographic differentiation of coevolutionary interactions, thereby elucidating the relative contribution of such factors to the ecological and evolutionary dynamics of interspecific interactions.

## Supplementary Material

Additional file 1 **Study sites used for the analysis of latitudinal gradient of the weevil's attacks on the Japanese camellia**. List of the localities used for the analyses shown in Fig. 1G-I. Click here for file

Additional file 2 **Nonlinear selection coefficients for camellia pericarp thickness**. Nonlinear selection coefficients for camellia pericarp thickness based on the data of the number of surviving seeds. Click here for file
