# Supplementary material for: Natural selection drives the fine-scale divergence of a coevolutionary arms race involving a long-mouthed weevil and its obligate host plant
Source: BMC Evol Biol. 2009 Nov 27;9:273. doi: 10.1186/1471-2148-9-273 (PMC2789073; doi:10.1186/1471-2148-9-273)
Supplement: Additional file 1 — Study sites used for the analysis of latitudinal gradient of the weevil's attacks on the Japanese camellia. List of the localities used for the analyses shown in Fig. 1G-I. [file 1471-2148-9-273-S1.PDF]

Additional file1. Study sites used for the analysis of latitudinal gradient of the weevil's attacks on the Japanese camellia. Data were obtained in 2003.

Locality	Latitude (°N)	Longitude (°E)	No. trees	No. fruits	No. trial holes	No. reaching holes	No. seeds	No. infested seeds
Notsumi	37.69	138.80	30	63	330	292	295	59
Kashiwazaki	37.33	138.47	24	27	229	166	109	46
Kutsuki*	35.37	135.91	25	44	1000	742	178	82
Hiratsuka	35.32	139.32	37	62	317	240	275	46
Kyoto*	35.02	135.81	21	23	566	361	73	29
Jurinji*	34.78	135.31	32	61	1927	1546	238	126
Nara*	34.69	135.88	37	86	1050	760	480	187
Shodoshima*	34.51	134.36	28	44	10	5	259	2
Kiikatsuura*	33.65	135.99	27	34	394	288	212	68
Taiji*	33.58	135.96	17	37	228	154	248	47
Arafune*	33.53	135.89	37	58	131	93	319	18
Kiioshima*	33.47	135.86	19	46	915	570	173	63
Usa*	33.43	133.46	33	50	327	152	316	25
Muroto*	33.25	134.19	37	55	384	206	310	62
Ashizuri*	32.73	133.03	67	102	2813	762	521	125
Reihoku*	32.53	130.03	39	62	2887	828	350	112
Takahama*	32.34	129.98	39	93	1107	434	619	99
Ushibuka*	32.16	130.03	10	18	0	0	122	0
Shimama	30.46	130.86	18	54	179	96	387	23
Yahazu*	30.46	130.50	41	101	2938	1178	512	141
Hanyama*	30.38	130.39	21	51	1025	172	365	56
Onoaida	30.24	130.55	9	34	311	36	270	8

*Data from a previous study [12]
