# Supplementary material for: Natural selection drives the fine-scale divergence of a coevolutionary arms race involving a long-mouthed weevil and its obligate host plant
Source: BMC Evol Biol. 2009 Nov 27;9:273. doi: 10.1186/1471-2148-9-273 (PMC2789073; doi:10.1186/1471-2148-9-273)
Supplement: Additional file 2 — Nonlinear selection coefficients for camellia pericarp thickness. Nonlinear selection coefficients for camellia pericarp thickness based on the data of the number of surviving seeds. [file 1471-2148-9-273-S2.PDF]

Additional file2. Nonlinear selection coefficients for camellia pericarp thickness. The number of surviving seeds, which was converted into relative fitness, was regressed on the pericarp thickness of camellia trees for each population (quadratic regression). The effect of tree size on the number of surviving seeds was controlled by incorporating diameter at breast height (DBH) as an explanatory variable. Both explanatory variables (i.e. pericarp thickness and DBH) were *z*-standardized (zero-mean, unit-variance) before regression. The partial regression coefficient for squared pericarp thickness was doubled to obtain standardized nonlinear selection coefficient (i.e. γ_σ) [60].

Locality	Variable	Coef.	SE	*t*	*P*
Fukagawa (FK)	$pericarp^2$	0.244	0.232	1.1	0.301
	pericarp	0.015	0.132	0.1	0.910
	DBH	0.002	0.121	0.0	0.990
Shiratani (SR)	$pericarp^2$	−0.133	0.316	−0.4	0.6774
	pericarp	0.538	0.193	2.8	0.0092
	DBH	0.139	0.190	0.7	0.4695
Kawahara (KW)	$pericarp^2$	−0.128	0.221	−0.6	0.565
	pericarp	−0.065	0.148	−0.4	0.662
	DBH	0.657	0.148	4.4	< 0.0001
Ohko-rindoh (OK)	$pericarp^2$	0.486	0.346	1.4	0.1711
	pericarp	0.715	0.274	2.6	0.0143
	DBH	0.205	0.277	0.7	0.4645
